# Nurses’ Job Insecurity and Emotional Exhaustion: The Mediating Effect of Presenteeism and the Moderating Effect of Supervisor Support

**DOI:** 10.3389/fpsyg.2020.02239

**Published:** 2020-09-18

**Authors:** Jihao Zhang, Shengnan Wang, Wei Wang, Geyan Shan, Shujie Guo, Yongxin Li

**Affiliations:** ^1^Institute of Psychology and Behaviour, Henan University, Kaifeng, China; ^2^Nursing Department, Henan Province People’s Hospital, Zhengzhou, China

**Keywords:** nurses, presenteeism behavior, job insecurity, emotional exhaustion, supervisor support

## Abstract

Presenteeism refers to attending work when one is ill, which not only leads to a decline in the physical and mental health of employees but also negatively impacts organizational productivity and increases an organization’s extra costs. Therefore, to explore the antecedents and outcomes of nurses’ presenteeism behavior and the acting mechanism among the variables, a sample of 330 nurses from China were investigated with the Sickness Presenteeism Questionnaire, Job Insecurity Scale, Perceived Supervisor Support Scale, and Emotional Exhaustion Scale. The results indicated that (1) job insecurity had a significantly predictive effect on nurses’ presenteeism behavior; (2) nurses’ presenteeism partially mediated the relationship between job insecurity and emotional exhaustion; and (3) supervisor support moderated the relationship between nurses’ presenteeism behavior and emotional exhaustion; i.e., the higher the level of supervisor support, the weaker the positive relationship between nurses’ presenteeism behavior and emotional exhaustion. The findings provide theoretical guidance and an empirical basis for prevention and intervention strategies concerning nurses’ presenteeism behavior.

## Introduction

Presenteeism behavior refers to the behavior of an employee who attends work despite feeling unwell and should be on sick leave (Aronsson et al., 2000). Employee presenteeism behavior is a common phenomenon in the workplace: studies show that 88% of employees in organizations have attended work while sick (McKevitt et al., 1997). Further, 65.6% of employees in Canadian government agencies had attended work while sick in 2005, and the average number of working days with illness per employee was 11.9 (Biron et al., 2006). In China, 74% of employees have had to work with an illness, and the average frequency is up to once per month (Li et al., 2019). Presenteeism is not only harmful to employees’ physical and mental health (Kivimaki et al., 2005; Taloyan et al., 2012), it also decreases organizational productivity and increases extra cost to organizations. According to a British survey in 2007, the annual cost of presenteeism to British companies is about 15.1 billion pounds (Parsonage, 2007). In 2010, American enterprises spent as much as 26 billion dollars owing to employees’ presenteeism behavior (Weaver, 2010).

Attributed to the nature of nursing work, such as a high workload, forced overtime, night shift work, and low substitutability (Martinez and Ferreira, 2012; Allemann et al., 2019), nursing is one of the occupations with a high incidence of presenteeism behavior (Bergstrom et al., 2009a,b; Yang et al., 2018). According to McKevitt et al. (1997), 85% of healthcare professions have experienced working despite an illness. It is urgent to discover the determinants of presenteeism behavior. Yang et al. (2016) found that stress-related factors at work are significantly correlated with presenteeism. Specifically, the hindrance stress, such as role ambiguity and interpersonal conflicts, can positively predict presenteeism, while the challenge stress, such as chances for learning and achievement, can reduce presenteeism *via* the mediation of health (Yang et al., 2018). Further, the organization’s supportive factors like coworker support and supervisor support can reduce presenteeism by increasing distributive justice (Yang et al., 2019) and reducing job stress (Yang et al., 2015).

Presenteeism behavior not only seriously affects nurses’ physical and mental health, resulting in decreased nursing quality and job satisfaction, which affects the treatment and rehabilitation of patients, but also brings direct and indirect economic losses to the organization (Letvak et al., 2012; Li et al., 2019; Schmidt et al., 2019). For example, Letvak et al. (2012) showed that nurse presenteeism behavior often leads to an increased number of medication errors and patient falls and subsequently decreases nursing quality and that the total annual cost of nurses’ presenteeism behavior in North Carolina is between $2 and $13 billion. Therefore, nurses’ presenteeism behavior has aroused the attention of researchers in the fields of mental health, nursing management, and public health and occupational health in North America and Europe (Virtanen et al., 2003; Pilette, 2005; Allemann et al., 2019).

However, most studies on presenteeism in the workplace have focused on European and Western countries (e.g., Britain, Sweden, the Netherlands, and the United States), and more empirical researches are still required to examine presenteeism behavior in an Eastern cultural context (Zhang and Li, 2016; Xi et al., 2019). Moreover, owing to the changing environment of economy and organizations, the increased work pressure and unemployment rate foster employees’ job insecurity (Cheng and Chan, 2008). As a coping strategy, the culture of overtime work has become prevalent in Chinese enterprises. Moreover, as the Chinese have always attached vast importance to work safety (Hofstede and Minkov, 2010), which is influenced by its Confucius culture, Chinese employees seem to be more likely to show more presenteeism behaviors. According to cross-cultural research conducted by Lu et al. (2013a), Chinese employees’ presenteeism behaviors are more common than those of British employees.

**Figure 1:**
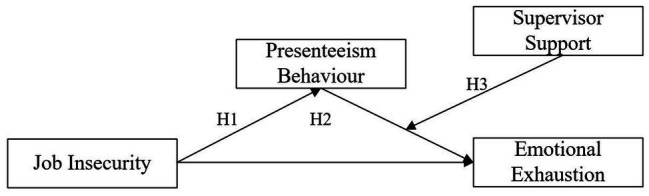
The moderated mediation model. Presenteeism behavior mediates the relationship between job insecurity and emotional exhaustion, and the second half of the mediation path is moderated by supervisor support.

In sum, it is necessary to determine the causes and effects of presenteeism behavior in Chinese organizations, which can provide a theoretical basis for effective human-centered management practice. Specifically, this study examined Chinese nurses to investigate the mediating effect of presenteeism behavior between nurses’ job insecurity and emotional exhaustion. Further, we explored the moderating effect of supervisor support between presenteeism behavior and emotional exhaustion. Therefore, we aimed to provide empirical evidence for prevention and intervention strategies that address nurses’ presenteeism behavior in the future. The moderated mediation model is shown in Figure 1.

### Job Insecurity and Presenteeism

As an important source of stress, job insecurity has a series of negative effects on employees’ work attitude and behavior (Cheng and Chan, 2008; Zhao et al., 2010). Job insecurity prompts employees to invest and transfer their resources when they perceive that their resources are threatened (Greenhalgh and Rosenblatt, 1984). When the exchange of resources is unbalanced, it will lead to negative consequences such as employees’ inability and finally results in resource exhaustion. According to the Conservation of Resources Theory (Hobfoll, 2011), the fear of job instability and sustainability has activated the process of resource consumption. To maintain one’s existing job position and working environment, employees will use psychological and emotional resources to alleviate the negative impact of job insecurity. When several resources are not paid back, employees will experience emotional exhaustion symptoms (Crawford et al., 2010). For example, Zhang et al. (2017b) reported that Chinese nurses’ job insecurity had a significant positive predictive effect on their emotional exhaustion.

Moreover, the level of job insecurity often affects whether an employee decides to work despite an illness (Johns, 2010). The frequency of employees’ presenteeism behavior is comparatively high after an organization’s downsizing. This is because, after the organization’s structural reform and downsizing, employees’ job insecurity will increase along with the workload and job competitiveness. Thus, to retain one’s job position and increase job stability, employees often prefer to work with an illness rather than take a rest or go to the hospital (Virtanen, 1994; Simpson, 1998). Hansen and Andersen (2008) confirmed that job insecurity can lead to an increase in the frequency of employees’ presenteeism behavior owing to the fear of job loss. Further, Bierla et al. (2013) showed a significantly positive correlation between job insecurity and presenteeism behavior because employees regard presenteeism as an effective strategy to reduce job insecurity, particularly when they believe that attending work despite an illness can highlight their loyalty and commitment to the organization and their commitment to work, which can ensure their job stability by structuring a strong psychological contract between employees, organizations, and leaders. Consequently, since the predictive effect of nurses’ job insecurity on emotional exhaustion has been supported, we proposed the following hypothesis:

*Hypothesis* 1: Job insecurity will significantly predict nurses’ presenteeism behavior.

### Mediation Effects of Presenteeism

Based on the Conservation of Resources Theory (Hobfoll, 2011), the total amount of an individual’s resources is limited, and once the resources are consumed, the individual needs time to have the resources recovered. Besides, according to Effort-recovery Theory, employees need enough time for physical and mental recovery after they have performed physical and mental work; if the recovery is insufficient, then the individuals’ psycho-physiological system remains active and cannot be restored to a self-equilibrium state (Meijman and Mulder, 1998). When employees are fatigued, they will have to make extra efforts during the following work period. Therefore, the accumulation of negative loads leads to further depletion of one’s energy and prolonged fatigue and eventual exhaustion (Sonnentag et al., 2012). Employees’ presenteeism behavior deprives individuals of the opportunity to recover from stress and illness, leading to increased fatigue, stress, and tension, and ultimately to emotional exhaustion. A longitudinal study conducted by Demerouti et al. (2009) found that there was a significant positive correlation between nurses’ presenteeism behavior and emotional exhaustion. Studies have also revealed that presenteeism behavior has a significant predictive effect on the emotional exhaustion of employees (Yildirim et al., 2014). Consequently, we proposed the following second hypothesis:

*Hypothesis* 2: Nurse presenteeism behavior will mediate the relationship between job insecurity and emotional exhaustion.

### Moderation Effects of Supervisor Support

Job Demand-Resource Model (Bakker and Demerouti, 2007) is proposed on the basis of the Conservation of Resources Theory (Hobfoll, 2011). It believes that each job position has two risk factors related to stress, namely, job demands and job resources (Bakker and Demerouti, 2007). Job demands are requirements for continuous physical and mental effort, involving physical, social, and organizational aspects of work, and are therefore often associated with physical or psychological consumption, while job resources involve material, psychological, social, and organizational resources, which can reduce work requirements and related physical and psychological efforts, contribute to the realization of work goals, and promote individual job development. Job resources not only can lead to high work engagement, low cynicism, and excellent job performance but also can reduce the harmful effect of work requirements on health (Bakker and Demerouti, 2007; Bakker and Evangelia, 2008). One key job resource – supervisor support – is significantly positively correlated with work engagement (Mauno et al., 2007) and can reduce the impact of work requirements on work pressure (Xanthopoulou et al., 2007). Supervisor support can foster employees’ needs for belongingness and being valued, thus motivating them to effectively cope with difficulties (e.g., physical discomfort at work). However, if employees work while sick, they often need extra attention and may seek more resources to complete their work effectively. Thus, supervisor support can weaken the relationship between presenteeism behavior and emotional exhaustion. Studies have shown that supervisor support can effectively eliminate the psychological stress of employees caused by work-related issues, such as role pressure (Babin and Boles, 1996) and psychological tension (O’Driscoll et al., 2003). Yang et al. (2015) found that supervisor support can negatively predict presenteeism through the decrease of job stress and the increase of distributive justice (Yang et al., 2019). A longitudinal study on IT workers conducted in Portugal proved that supervisor support can buffer the negative impact of presenteeism on productivity *via* reducing role ambiguity (Zhou et al., 2016). Thus, we proposed the following third hypothesis:

*Hypothesis* 3: Supervisor support will moderate the relationship between presenteeism behavior and emotional exhaustion.

## Materials and Methods

### Participants

In this study, 330 in-service nurses (from a pool of 370 nurses; response rate = 89.2%) from comprehensive hospitals in Henan province, China, were selected as participants. All participants were informed of the study purpose and the confidentiality principles before the survey; then, anonymous surveys were administered in each department after participants provided informed consents. All participants were women; consistently, most nurses in China are women. Participants’ mean age was 28 years (*SD* = 4.51); 142 (43%) were unmarried and 188 (57%) were married; 27 (8.2%) were unregistered nurses, 65 (19.7%) were nurses, 174 (52.7%) were senior nurses, and 64 (19.4%) were chief nurses. Among the 330 participants, 256 (77.6%) participants had college degrees, and 74 (22.4%) participants did not. Of them, 191 (57.9%) participants had work as a nurse less than 5 years, 83 (25.2%) participants had work for 6–10 years, and 58 (17.6%) participants had a tenure over 11 years.

### Measures

#### Sickness Presenteeism Questionnaire

The presenteeism behavior questionnaire developed by Lu et al. (2013b) was adopted to measure the incidence of presenteeism. The questionnaire asked participants to rate the number of times in the past 6 months that they had done the following: “Although you feel sick, you still force yourself to go to work” and “Although you have physical symptoms such as a headache or backache, you still force yourself to go to work.” Responses were measured with a four-point Likert scale: 1 = *never*, 2 = *once*, 3 = *two to five times*, and 4 = *more than five times*. Higher scores indicated a higher frequency of presenteeism behavior. In this study, Cronbach’s alpha was 0.84.

#### Job Insecurity Scale

The job insecurity scale by Hellgren et al. (1999) was adopted in this study. The scale consists of seven items, including two dimensions: job quantity insecurity (three items; e.g., “I feel uneasy about losing my job in the near future”) and job quality insecurity (four items; e.g., “My pay development in this organization is promising”). Some items were reverse scored. Responses were measured with a five-point Likert scale, ranging from 1 (*strongly disagree*) to 5 (*strongly agree*). Higher scores indicated stronger job insecurity. In this study, Cronbach’s alpha of the total scale and each dimension were 0.67, 0.66, and 0.83, respectively. This scale has been widely used and is considered reliable and valid (Wang et al., 2016).

#### Perceived Supervisor Support Scale

The supervisor support scale developed by Zhou (2009) was adopted. The scale contains 15 items, including three dimensions of work support (seven items; e.g., “my boss is very helpful when I have problems in my work”), employee value identity (three items; e.g., “my boss is proud of his/her employees’ achievements”), and the relationship between interests (five items; e.g., “my boss considers the interests of employees when making decisions”). Responses were measured with a five-point Likert scale, ranging from 1 (*strongly disagree*) to 5 (*strongly agree*). Higher scores indicated stronger supervisor support as perceived by employees. In this study, Cronbach’s alpha of the total scale and each dimension were 0.96, 0.93, 0.79, and 0.90, respectively.

#### Emotional Exhaustion Scale

The emotional exhaustion subscale of the job burnout scale created by Li (2003) was adopted. The scale contains five items, such as “I often feel exhausted.” All items were scored on a seven-point Likert scale, ranging from 1 (*completely inconsistent*) to 7 (*completely consistent*). Higher scores indicated stronger emotional exhaustion. In this study, Cronbach’s alpha was 0.77.

### Data Analysis

SPSS 22.0 and AMOS 20.0 were utilized to analyze the collected data. A confirmatory factor analysis and homogeneity reliability analysis were conducted to examined common method bias. Product difference correlation was used to explore the correlations among variables. A hierarchical regression analysis with a bias-corrected bootstrap technique was conducted to investigate the mediating effect of presenteeism behavior in the relationship between job insecurity and emotion exhaustion and the moderating effect of supervisor support in the relationship between presenteeism behavior and emotional exhaustion.

Moreover, the common method variance was calculated according to the Podsakoff et al. (2003). The criteria were 25% median score (Williams et al., 1989) and the change of *χ*^2^ (Richardson et al., 2009). The other statistical tests followed the significance coefficient (i.e., 5%), which is generally accepted in psychology (e.g., Yoshida, 2004).

## Results

### Common Method Bias Test

Following the suggestion of Podsakoff et al. (2003), the unmeasured latent methods factor was also applied. On the basis of the original four-factor structure (i.e., items of presenteeism, job insecurity, perceived supervisor support, and emotional exhaustion loading on their respective construct), a latent method factor (common method variance) was also constructed, and all items were allowed to load on it. The latent factor was uncorrelated with other factors. The variance explained by the latent method factor was 4%, which is lower than the 25% median score in published studies (Williams et al., 1989). Furthermore, when constraining the latent method factor’s regression weight to 0, or setting estimation as free, the model fit does not change significantly (Δ*χ*^2^ = 22.76, n.s.; Richardson et al., 2009). These results provide further evidence that common method variance had little effect on the present study’s overall results.

### Descriptive Statistics and Correlation Analysis of Research Variables

Table 1 presents the descriptive statistics and correlation matrix of each research variable. Job insecurity was significantly positively correlated with presenteeism behavior (*r* = 0.24, *p* < 0.01) and emotional exhaustion (*r* = 0.22, *p* < 0.01). There was also a significant positive correlation between presenteeism behavior and emotional exhaustion (*r* = 0.31, *p* < 0.01). Supervisor support was negatively correlated with emotional exhaustion (*r* = −0.17, *p* < 0.01).

**Table 1:** Descriptive statistics and correlation analysis of research variables (*N* = 330).

Variable	*M* ± *SD*	1	2	3	4	5	6	7
Age	1.88 ± 0.71	1.00						
Marital status	1.57 ± 0.50	0.63^**^	1.00					
Job position	2.83 ± 0.83	0.77^**^	0.57^**^	1.00				
Job insecurity	2.90 ± 0.58	0.05	0.08	0.12^*^	1.00			
Presenteeism behavior	3.24 ± 0.73	0.13^*^	0.15^**^	0.12^*^	0.24^**^	1.00		
Emotional exhaustion	4.21 ± 1.27	0.07	0.11^*^	0.18^**^	0.22^**^	0.31^**^	1.00	
Supervisor support	4.17 ± 0.73	−0.07	−0.07	−0.05	−0.17^*^ ^*^	−0.01	−0.17^**^	1.00

*N* = 330; age: 1 = “≤25,” 2 = “26–30,” 3 = “≥31.” Marital status: 1 = unmarried, 2 = married

* *p* < 0.05

** *p* < 0.01.

### Test of the Moderated Mediation Model

**Figure 2:**
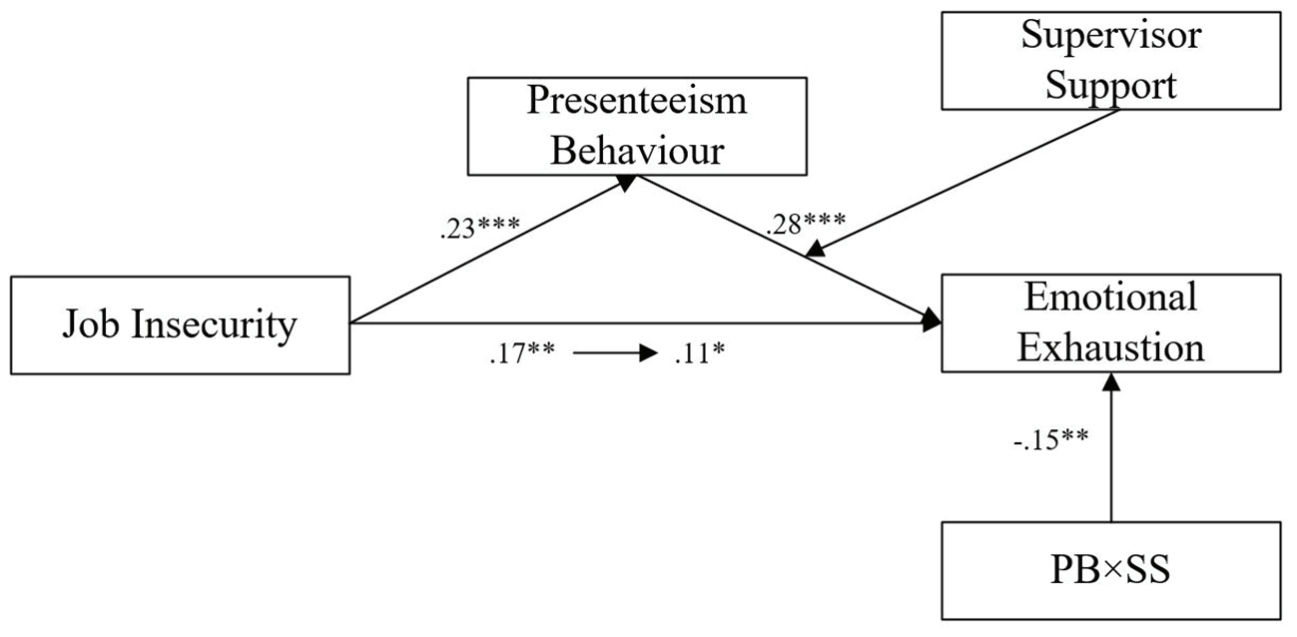
The moderated mediation model (with findings). Presenteeism behavior mediates the relationship between job insecurity and emotional exhaustion, and the second half of the mediation path is moderated by supervisor support. PB = Presenteeism behavior, SS = Supervisor support.

The moderated mediation model test was conducted through the method recommended by Wen and Ye (2014). First, the independent variable (*X*), dependent variable (*Y*), mediation variables (*W*), and regulation variables (*U*) were standardized into *Z*-scores (variable names unchanged); then, we multiplied the corresponding *Z*-scores to produce the interaction term *UW* scores and controlled for the effects of demographics variables on the results. The results are shown in Table 2 and Figure 2.

**Table 2:** Test of the moderated mediation model (*N* = 330).

Variable	Equation 1 (Criterion: EE)		Equation 2 (Criterion: PB)		Equation 3 (Criterion: EE)	
	*β*	*t*	*β*	*t*	*β*	*t*
Age	−0.17	1.94	0.07	0.84	−0.19	2.22^*^
Marital status	0.05	0.81	0.09	1.41	0.04	0.63
Job position	0.25	2.94^**^	−0.02	0.24	0.23	2.94^**^
Job insecurity	0.17	3.20^**^	0.23	4.36^***^	0.11	2.09^*^
Supervisor support	−0.13	2.58^*^	0.04	0.78	−0.15	2.94^**^
Presenteeism behavior					0.28	5.36^***^
PB × SS					−0.15	3.13^**^
*R*^2^	0.10		0.07		0.19	
*F*	7.17^***^		5.58^***^		10.83^***^	

* *p* < 0.05

** *p* < 0.01

*** *p* < 0.001.

In Equation 1, job insecurity had a significant positive predictive effect on emotional exhaustion (*β* = 0.17, *t* = 3.20, *p* < 0.01), indicating that job insecurity can promote emotional exhaustion. In Equation 2, job insecurity had a significant positive predictive effect on presenteeism behavior (*β* = 0.23, *t* = 4.36, *p* < 0.001), indicating that the higher the level of job insecurity perceived by nurses, the higher the frequency of presenteeism behavior. In Equation 3, presenteeism behavior had a significant positive predictive effect on emotional exhaustion (*β* = 0.28, *t* = 5.36, *p* < 0.001), indicating that the higher the frequency of nurses’ presenteeism, the higher the level of perceived emotional exhaustion. Therefore, presenteeism mediated the effect of job insecurity on emotional exhaustion. Furthermore, in Equation 3, the effect of job insecurity on emotional exhaustion remained significant (*β* = 0.11, *t* = 2.09, *p* < 0.05), which indicates that presenteeism behavior partially mediated the association between job insecurity and emotional exhaustion. Additionally, in Equation 3, the interaction of presenteeism behavior with supervisor support had a significant negative predictive effect on emotional exhaustion (*β* = −0.15, *t* = 3.13, *p* < 0.01). Thus, supervisor support moderated the relationship between nurse presenteeism behavior and emotional exhaustion. In other words, supervisor support moderated the mediation path of job insecurity to presenteeism behavior and emotional exhaustion. Based on the above results, the moderated mediation model proposed in this study was verified.

The conditional indirect effect test program of Preacher et al. (2007) was used to further analyze the effect size and confidence interval of the moderated mediation model. According to the no. 14 model in PROCESS, the non-parametric percentile bootstrap method was used to conduct parameter estimation. The results are shown in Table 3. When supervisor support was one standard deviation below the mean value, the mediating effect of presenteeism behavior between job insecurity and emotional exhaustion was moderated by supervisor support. The mediating effect was 0.10, accounting for 51.8% of the total effect. That means that when the level of supervisor support is low, the indirect effect increases and the direct effect decreases; that is, job insecurity increases employees’ emotional exhaustion by increasing the frequency of their presenteeism behavior. When supervisor support was at the mean level (*U* = 0), the mediating effect of job insecurity on emotional exhaustion was moderated by supervisor support through presenteeism behavior. The mediating effect was 0.06, accounting for 33.2% of the total effect, which means the indirect effect of job insecurity on emotional exhaustion through presenteeism accounts for about one third of the total effect. When supervisor support was one standard deviation above the mean value, the mediating effect of job insecurity on emotional exhaustion was moderated by supervisor support through presenteeism behavior. The mediating effect was 0.02, accounting for 14.6% of the total effect. This indicates that when supervisor support was high, the indirect effect decreased, and the direct effect increased. In other words, although job insecurity increased the frequency of nurses’ presenteeism behavior, it did not increase their emotional exhaustion level.

**Table 3:** Mediating effect and confidence interval at different levels of the moderating variable (*N* = 330).

Mediator	Moderator: supervisor support	Effect	*SE*	Bootstrap (95% CI)
Presenteeism behavior	*M* − *SD*	0.10	0.03	(0.04, 0.17)
Presenteeism behavior	*M*	0.06	0.02	(0.02, 0.11)
Presenteeism behavior	*M* + *SD*	0.02	0.01	(0.00, 0.07)

**Figure 3:**
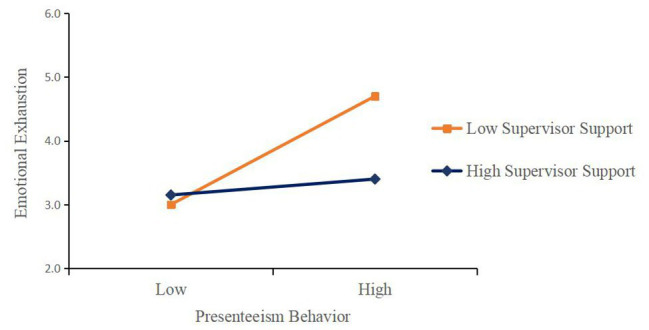
Simple slope analysis.

To show the moderating effect of supervisor support more intuitively, the effects of nurses’ presenteeism behavior on emotional exhaustion were analyzed under the condition of high–low supervisor support. In this study, supervisor support was divided into a high group (*M* + 1 *SD*) and a low group (*M* − 1 *SD*), and a simple slope test was conducted. As shown in Figure 3, the effect of nurses’ presenteeism behavior on emotional exhaustion increased with the growth of the frequency of the behavior, regardless of the level of supervisor support. Therefore, the level of supervisor support did not change the direction of the effect of nurses’ presenteeism behavior on emotional exhaustion. Specifically, under the condition of low supervisor support, nurses’ presenteeism behavior had a significant predictive effect on emotional exhaustion (*β* = 0.60, *t* = 4.91, *p* < 0.001); that is, for the nurses with low supervisor support, emotional exhaustion significantly increased with increased presenteeism behavior. However, for nurses with high supervisor support, the predictive effect of nurse presenteeism behavior on emotional exhaustion was non-significant (*β* = 0.16, *t* = 1.30, *p* > 0.05), indicating that high supervisor support significantly weakened the relationship between nurse presenteeism behavior and emotional exhaustion.

## Discussion

This study examined 330 nurses from Henan province to investigate the relationship between job insecurity and emotional exhaustion *via* the mediating effects of presenteeism behavior and the moderating effects of supervisor support. The results suggest that job insecurity has a significant positive predictive effect on emotional exhaustion, that nurse presenteeism behavior plays a partial mediating role between job insecurity and emotional exhaustion, and that supervisor support moderates the relationship between nurse presenteeism behavior and emotional exhaustion.

The results of correlation analyses showed that job insecurity is positively correlated with emotional exhaustion. This result is consistent with the results of Zhang et al. (2017a); that is, job insecurity – an important source of stress – negatively impacts individuals’ physical and mental health to some extent. On the one hand, when nurses feel their work stability is threatened, they may increase their working time and working intensity to reduce the insecurity caused by workload. However, if their efforts go unrewarded, emotional exhaustion occurs. In contrast, according to the conservation of resource theory (Hobfoll, 1989), when nurses feel that their career development opportunities (e.g., raises and promotion) are limited and their working environment is poor, they will be stimulated to protect their own resources. They will invest a lot of psychological, physiological, and emotional resources to maintain and protect the resources that are vital to them. This may result in their limited resources becoming exhausted, resulting in a lack of passion for work, loss of work achievement, and eventually emotional exhaustion.

The same result was found concerning nurses’ presenteeism behavior, which partially mediated the relationship between job insecurity and emotional exhaustion. When nurses come to work with an illness, they must expend extra physical, psychological, and emotional resources. However, as being deeply influenced by Confucian culture that regards “diligence” as one of the precious qualities of employees, Chinese nurses also strive to be diligent in their work (Lu et al., 2013a). Therefore, even when they are ill, they are likely to choose to be at work whenever possible. According to the conservation of resource theory (Hobfoll, 2011), individuals’ resources are limited. When resources are consumed for a long time without recovery, it leads to increased levels of fatigue, anxiety, and tension in individuals, as well as negatively affects the productivity of an organization.

Supervisor support moderated the mediating process between job insecurity and emotional exhaustion of nurse presenteeism behavior, and the mediating effect was mainly reflected in the second half of the mediating chain; that is, the relationship between nurse presenteeism behavior and emotional exhaustion depended on the level of supervisor support. Specifically, job insecurity will lead to an increase in the frequency of nurse presenteeism behavior. Compared with nurses with high supervisor support levels, nurses with low supervisor support levels displayed higher levels of emotional exhaustion.

Consistent with past research, supervisor support can reduce the influence of job demands on work pressure (Xanthopoulou et al., 2007), improve employees’ work engagement (Mauno et al., 2007), and promote employees’ sense of belongingness and self-worth. These motivate nurses to cope with difficulties; thus, supervisor support may alleviate the negative effects caused by presenteeism behavior. Specifically, presenteeism behavior deprives nurses of the opportunity to recover from stress and illness (Meijman and Mulder, 1998); causes increased fatigue, stress, and workload; and will lead to emotional exhaustion if effective supervisor support is not obtained. However, high supervisor support can help alleviate the adverse consequences of presenteeism behavior and offset the risk of emotional exhaustion. In sum, our results suggest that job insecurity increases the frequency of nurses’ presenteeism behavior, but high supervisor support significantly reduces the emotional exhaustion caused by nurses’ presenteeism behavior.

### Theoretical Implications

Presenteeism behavior is widely existing in workplaces and attracts great attention from scholars. Increasing number of scholars have invested in this field and attempted to catch the antecedents of presenteeism. These findings pointed out that job insecurity is an essential influential factor of presenteeism behavior. Meanwhile, over decades, scholars have attempted to discover the causes of emotional exhaustion in workplaces. As job insecurity has been confirmed as an important antecedent (Zhang et al., 2017b), it is necessary to identify the possible working mechanisms between the two variables. Therefore, the present findings contribute to reveal one of the working mechanisms of emotional exhaustion caused by job insecurity. Moreover, this study identified presenteeism behavior and its outcome and investigated the role of presenteeism behavior in the relationship between job insecurity and emotional exhaustion, which makes the relationship between these two variables clearer. Furthermore, compared with the large number of researches conducted in Europe and North America (Virtanen et al., 2003; Pilette, 2005; Martinez and Ferreira, 2012; Allemann et al., 2019), this research helps to enrich the presenteeism literature with eastern cultural background by adding empirical data of a divergent population from China. Besides, the present study also contributes to span the application scope of the Conservation of Resources Theory (Hobfoll, 2011). Although the Conservation of Resources Theory (Hobfoll, 2011) has been widely used in many fields, this study brings a modest but significant contribution to the related literature. Additionally, this research extends the presenteeism literature by building a new integrated framework, in which presenteeism was positively related to job insecurity and emotional exhaustion, and partially mediated the relationship, while perceived supervisor support moderated the relation of presenteeism and emotional exhaustion. Despite that, the framework was confirmed with the empirical data, and all the hypotheses proposed in this study were supported.

### Practical Implications

Through an empirical study of nurses’ presenteeism in the workplace, this study discusses its negative consequences and mechanism, which has important practical value for medical policymakers and nurses. First, medical and health management departments should pay attention to the prevention of nurses’ presenteeism behavior. They should make great efforts to construct a harmonious working environment; formulate reasonable salary distribution and a promotion system; provide more training, education, and professional development opportunities; improve the doctor–patient relationship; and ease nurses’ concerns regarding the risk of job loss to deter nurses’ job insecurity and the frequency of presenteeism behavior. Giving nurses ample opportunities to recover from illness and stress will improve their work efficiency and reduce the costs to the organization. Second, supervisors should provide more support, incentives, and help to their subordinates to develop their individual resilience, which will improve their ability to cope with challenges and reduce the harmful effects of presenteeism. Third, nurses should be aware of the dangers of presenteeism behavior on their health, work attitude, and work performance. Effectively reducing presenteeism behavior may improve nurses’ work efficiency, thus allowing them to provide better services for patients’ rehabilitation and treatment.

### Limitations and Future Research

This study had some limitations. The first limitation is that the cross-sectional design limits our ability to infer causality or determine changes in participants’ presenteeism behaviors over time. Therefore, longitudinal studies are suggested to investigate whether nurses’ presenteeism behaviors have continuous negative effects on outcome variables and the interaction between variables (Ruhle et al., 2020). Second, the local data and small sample size limit the generalization of the conclusion. It is suggested to employ more participants with diversified backgrounds in future research. Third, this study mainly adopted self-reported methods to measure the variables and collect the data. Assessing individuals’ subjective experiences is always prone to common method bias. Therefore, in future research, data sources should be multifarious and diversified survey methods should be adopted. For example, data could be collected by self-reported scales combined with other-reported measures, as well as by obtaining nurses’ daily work records from colleagues and direct supervisors.

## Conclusion

This study emphasized on the role of nurses’ presenteeism in the relationship of job insecurity and emotional exhaustion. The results have shown that the nurses’ job insecurity can positively predict presenteeism behavior, which means the higher level of job insecurity perceived by nurses, the higher possibility for them to commit presenteeism behavior. The results not only proved the mediating role of presenteeism behavior between nurses’ job insecurity and emotional exhaustion but also confirmed that supervisor support could weaken the negative impact of presenteeism behavior on emotional exhaustion. The study results contribute to enrich the literature of relevant variables and contribute to the Conservation of Resources Theory. As nurses’ presenteeism behavior can positively predict the emotional exhaustion at work, the management department is suggested to pay more attention to this issue, making greater efforts to prevent the occurrence of this behavior. Further, it is necessary to strengthen supervisor support to reduce the negative impact of presenteeism on nurses’ emotional exhaustion.

## Data Availability Statement

The datasets generated for this study are available on request to the corresponding author.

## Ethics Statement

The studies involving human participants were reviewed and approved by Henan University Institutional Review Board. The patients/participants provided their written informed consent to participate in this study.

## Author Contributions

YL is the principal investigator for the study, generated the idea, and designed the study. JZ and SW were the primary writers of the manuscript and approved all changes. WW and GS supported the data input and data analysis. SG supported the data collection. All authors were involved in developing, editing, reviewing, and providing feedback for this manuscript and have given approval of the final version to be published.

### Conflict of Interest

The authors declare that the research was conducted in the absence of any commercial or financial relationships that could be construed as a potential conflict of interest.
